# Dual targeting of SLC6A14 and autophagy/macropinocytosis enhances therapeutic efficacy in pancreatic ductal adenocarcinoma

**DOI:** 10.1042/BCJ20250155

**Published:** 2026-07-02

**Authors:** Mosharaf Mahmud Syed, Devaraja Rajasekaran, Souad R. Sennoune, Tanima Sharker, Oscar Sanchez, Mary Katherine Jurek, Longfa Kou, Ruijie Chen, Vadivel Ganapathy, Yangzom D. Bhutia

**Affiliations:** 1 Department of Cell Biology and Biochemistry, Texas Tech University Health Sciences Center, Lubbock, TX 79430, U.S.A.; 2 Department of Psychiatry, Tufts Medical Center, 800 Washington St., Boston, MA 02111, U.S.A.; 3 Department of Pharmacy, Wenzhou Municipal Key Laboratory of Pediatric Pharmacy, The Second Affiliated Hospital and Yuying Children's Hospital of Wenzhou Medical University, Wenzhou 325027, China

**Keywords:** Alpha-methyl-l-tryptophan, autophagy, hydroxychloroqine, Macropinocytosis, Pancreatic ductal adenocarcinoma, SLC6A14

## Abstract

Pancreatic ductal adenocarcinoma (PDAC) is highly desmoplastic and undergoes metabolic reprogramming to sustain its growth and proliferation. Our laboratory has identified SLC6A14, an amino acid transporter, as a novel drug target for PDAC. Genetic deletion of SLC6A14 or its pharmacological blockade with α-MLT attenuates PDAC growth by inducing amino acid deprivation. However, nutrient stress, particularly amino acid deprivation, can induce nutrient scavenging mechanisms like autophagy and macropinocytosis, thereby undermining the full anticancer potential of SLC6A14 blockade. To address this, the current work was conducted to test whether SLC6A14 blockade induces autophagy and/or macropinocytosis and to further investigate whether dual inhibition of SLC6A14 (α-MLT) and autophagy/macropinocytosis (HCQ) would yield a better therapeutic outcome in PDAC as opposed to targeting SLC6A14 alone. *In vitro* assays (MTT and colony formation) revealed that the combination treatment significantly reduced PDAC cell viability and clonogenic potential as opposed to monotherapy. The treatment model subcutaneous xenograft in athymic nude mice demonstrated a superior therapeutic outcome with the combination regimen. Collectively, our study demonstrates that the afore-described combination therapy creates a metabolic trap wherein α-MLT induces nutrient stress, while HCQ prevents autophagic and macropinocytosis compensation, thus culminating in a more potent tumor attenuation. This dual blockade represents a hitherto unexplored treatment strategy for PDAC.

## Introduction

Pancreatic ductal adenocarcinoma (PDAC) has a highly desmoplastic stroma and therefore undergoes metabolic reprogramming to survive in a nutrient-austere microenvironment [1–4]. Amino acids, both essential and non-essential, are indispensable nutrients, and cancer cells utilize them for protein synthesis, energy production, one-carbon metabolism, and nucleotide/DNA synthesis for their continued growth and proliferation [5–7]. To meet their increased amino acid demands, cancer cells up-regulate specific amino acid transporters (AATs) to maintain the intracellular amino acid pools and support growth under nutrient-starved conditions. AATs such as SLC1A5/ASCT2, SLC7A5/LAT1, SLC7A11/xCT, and SLC38A5/SN2 are up-regulated in tumor cells and promote growth by supplying amino acids and activating tumor-promoting pathways such as mammalian target of rapamycin complex 1 (mTORC1) [8–11]. Among them, SLC6A14/ATB^0,+^ is unique since it is a sodium–chloride coupled AAT, with a broad substrate selectivity and the ability to highly concentrate the substrate inside the tumor cells. Consistent with this role, SLC6A14 has been reported to be significantly up-regulated in multiple malignancies. More interestingly, pharmacological blockade of SLC6A14 is known to induce amino acid starvation, inhibition of mTORC1 signaling, and up-regulation of LC3B, possibly indicating induction of autophagy [12–15].

In addition to transporter-mediated uptake of amino acids, cancer cells can activate nutrient scavenging pathways such as macroautophagy (hereafter referred to as autophagy) and macropinocytosis [16–21] during periods of nutrient stress, particularly amino acid deprivation. Autophagy is a lysosome-dependent catabolic pathway that recycles intracellular macromolecules to generate substrates during periods of nutrient stress. In contrast, macropinocytosis enables cells to internalize extracellular proteins and other macromolecules, which are subsequently degraded in lysosomes to release amino acids and other nutrients. Though autophagy and macropinocytosis are distinct processes, they operate in a complementary manner. In PDAC, both pathways are basally activated due to desmoplasia, hypoxic, and oncogenic KRAS signaling, which are further amplified under the stress of amino acid deprivation, thereby supporting tumor growth and survival [22–27].

Literature evidence has shown that amino acid deprivation or inhibition of certain AATs activates autophagy to compensate for the nutrient loss [9,28–30]. However, it remains unclear whether SLC6A14 blockade also activates these adaptive mechanisms in PDAC. Therefore, it is important to investigate whether these pathways are activated following SLC6A14 blockade, as they could restore intracellular amino acid pools, reactivate mTORC1 signaling, and thereby limit the therapeutic benefit of targeting this transporter.

In the present study, we tested the hypothesis that PDAC cells induce autophagy and/or macropinocytosis to compensate for amino acid loss induced by SLC6A14 blockade and that simultaneous inhibition of these adaptive pathways enhances therapeutic efficacy. We used alpha-methyl-l-tryptophan (α-MLT), a pharmacological blocker of SLC6A14, in combination with hydroxychloroquine (HCQ), a lysosomal inhibitor that disrupts both autophagy and macropinocytosis-mediated nutrient flux. Using *in vitro* studies as well as *in vivo* xenograft studies in athymic nude mice, we investigated whether dual inhibition of SLC6A14 and autophagy and macropinocytosis provides superior therapeutic benefit in PDAC as opposed to targeting SLC6A14 alone.

## Results

### SLC6A14 promotes PDAC and affects overall patient survival

**Figure 1:**
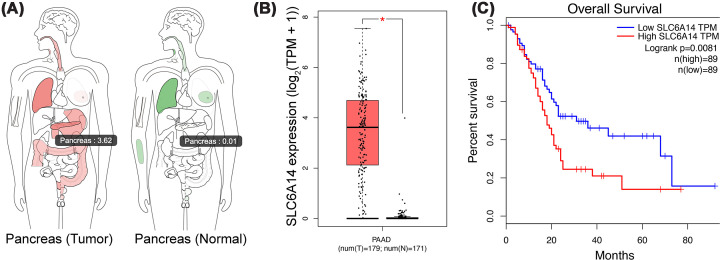
SLC6A14 is highly up-regulated in PDAC and the higher expression is associated with decreased overall patient survival (**A**) Interactive body map showing median SLC6A14 expression in PDAC tumor versus normal pancreas (Scale: Log_2_(TPM +1); (**B**) Box plot map showing SLC6A14 expression in PDAC tumor [tumor (T), *n* = 179; normal (N), *n* = 171] versus normal pancreas; (**C**) Kaplan–Meier curve showing survival probability between high and low SLC6A14 expression. Data are given as mean ± SEM. *P <0.05.

SLC6A14, an AAT with a broad substrate selectivity, is known to be significantly up-regulated in many solid tumors like colorectal cancer, estrogen-receptor-positive breast cancer, cervical cancer, and PDAC [31–34]. Using *in vitro* cell line models as well as *in vivo* mouse models (athymic nude mice and KPC spontaneous mouse model of PDAC), we have established the tumor-promoting role of SLC6A14 [12,35]. Here, we show the expression profile of SLC6A14 in 179 human PDAC tumors versus 171 normal human pancreases using the web-based tool, GEPIA (Gene Expression Profiling Interactive Analysis). The interactive body map as well as the box plot describing quantitatively the expression levels clearly show significant up-regulation of SLC6A14 expression in PDAC tumors as opposed to normal pancreases (Figure 1A,B). Furthermore, we also found a reciprocal relationship between SLC6A14 expression levels and survival probability; the higher the SLC6A14 expression, the lower is the survival probability in PDAC patients (Figure 1C). Therefore, based on our own published data and the data available online, SLC6A14 is a bonafide tumor promoter affecting overall patient survival.

### SLC6A14 blockade induces autophagy in PDAC cell lines and KPC mouse pancreas

**Figure 2:**
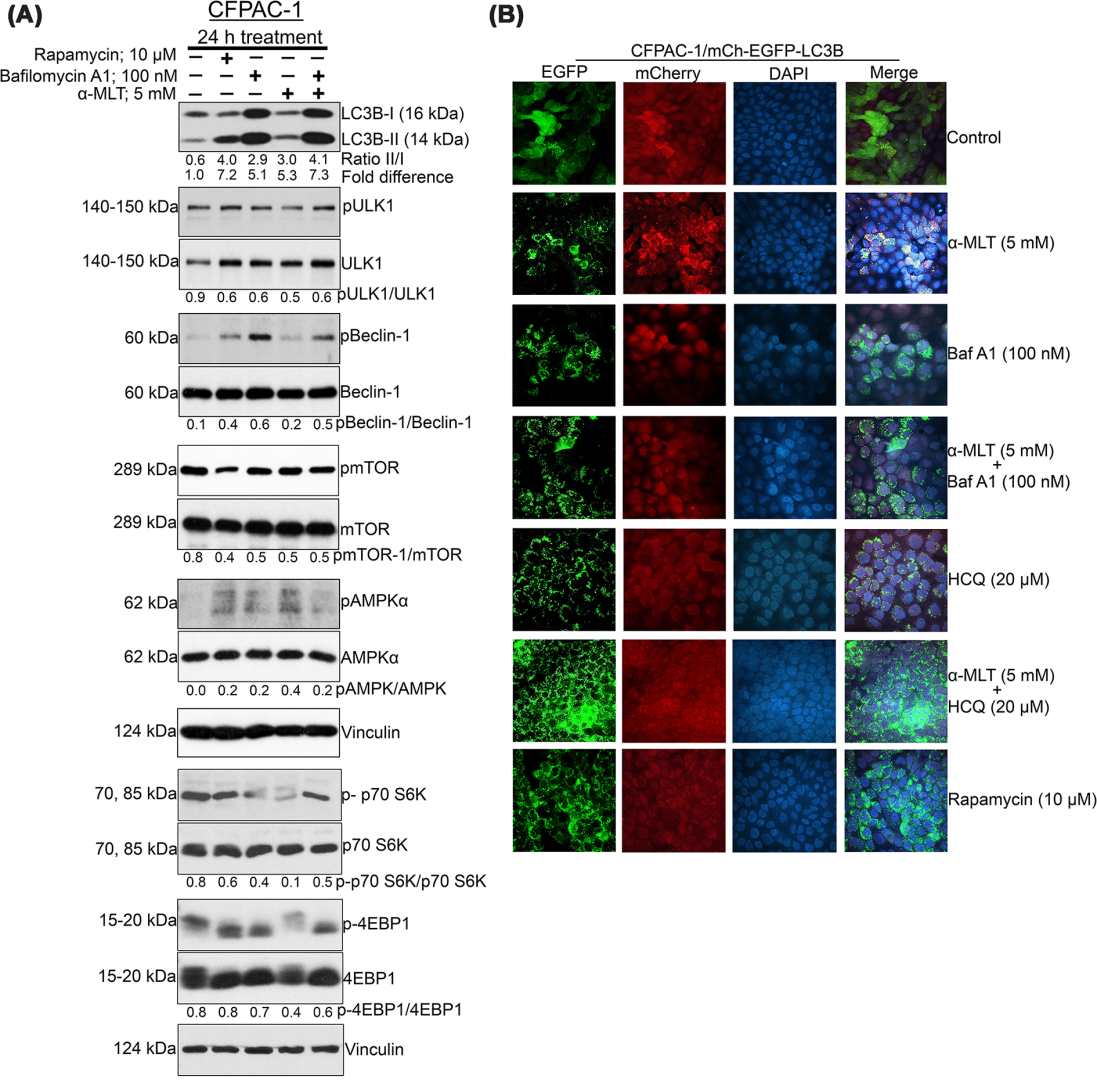
SLC6A14 blockade induces autophagy in PDAC cells (**A**) Western blot showing levels of LC3B, its upstream target proteins, and mTORC1 downstream effector proteins in CFPAC-1 cells. Rapamycin, an mTORC1 inhibitor, was used as a positive control; BafA1, an autophagy inhibitor, was used to assess flux, and α-MLT was used as a blocker of SLC6A14. Protein bands were quantified by densitometric analysis using ImageJ software. Normalized protein expression was calculated for each target, and the data are presented as the ratio of phosphorylated to the total protein. (**B**) Confocal microscopy in CFPAC-1/mCherry-EGFP-LC3B cells showing EGFP or mCherry signal following treatment with α-MLT, either singly or in combination with BafA1 and HCQ. Image magnification: 60×.

Autophagy is a nutrient scavenging mechanism that is known to be up-regulated in PDAC [36–43]. Our preliminary data using α-MLT indicated that the blockade of SLC6A14 induces autophagy in PDAC cells, further accentuating the basal level. SLC6A14 is an AAT, and therefore its blockade leads to amino acid starvation, inhibition of the mammalian target of rapamycin complex 1 (mTORC1), and ultimately tumor attenuation. However, if SLC6A14 blockade induces autophagy as a compensatory mechanism, this can undermine its full anticancer potential. Therefore, to fully understand whether SLC6A14 blockade induces autophagy or not, we performed Western blotting in SLC6A14-positive CFPAC-1 cells. LC3 is the most widely used autophagosome marker since the amount of LC3-II reflects the number of autophagosomes and autophagy-related structures. Nutrient deprivation in the form of serum and/or amino acids increases the number of autophagosomes, and, accordingly, the amount of LC3-II also increases [44,45]. However, it should be noted that the LC3-II amount at a given time point does not necessarily estimate the autophagic activity, since autophagy activation as well as inhibition of autophagosome degradation greatly increase the amount of LC3-II. Therefore, to measure the autophagic flux, it is essential to determine how much of LC3-II is degraded in a lysosome-dependent manner during a certain time point [46,47]. Bafilomycin A1 (BafA1), a potent V-ATPase inhibitor, is used to determine the lysosome-dependent degradation. The difference in the amount of LC3-II between samples with and without BafA1 represents the level of autophagic flux. Therefore, to study the autophagic flux, we treated CFPAC-1 cells with 5 mM α-MLT, either in the presence or absence of 100 nM BafA1, for 24 h. It was interesting to see that α-MLT treatment increased LC3-II levels (5.3-fold increase) when compared with the untreated control; however, this induction in LC3-II was further enhanced upon addition of BafA1 (7.3-fold increase), clearly indicating that SLCA614 inactivation induces autophagy in PDAC cells (Figure 2A). Rapamycin (10 μM), which is an inhibitor of mTORC1, was used as a positive control. Since autophagy and mTORC1 are closely associated, wherein the activation of one inhibits the other and vice versa [48,49], rapamycin treatment should induce autophagy, which is clearly evident in the immunoblotting. mTORC1, AMPK, ULK1, and Beclin-1 are the upstream regulators of autophagy, and therefore, if autophagy is induced following SLC6A14 blockade, mTORC1 should be inactivated, whereas AMPK and Beclin-1 should be activated [50,51]. Our data do show a decrease in the phosphorylation of mTORC1 at S2448 and an increase in phosphorylation of AMPK and Beclin-1 when compared with the untreated control. We also checked the phosphorylation status of p70 S6K1 and 4EBP1, the key downstream effectors of mTORC1 signaling, and the results were consistent with the observed changes in mTORC1 activity. These are the signature characteristics of autophagic induction; taken together the data show that SLC6A14 blockade elevates autophagic flux, indicating autophagic induction.

To further corroborate our immunoblotting results, confocal microscopy was performed in CFPAC-1 cells stably expressing the tandem fluorescent-tagged autophagy reporter mCherry-EGFP-LC3B. Because EGFP (pKa = 5.9) fluorescence is quenched in acidic compartments, while mCherry (pKa = 4.5) fluorescence remains relatively stable, the construct enables visualization of autophagic flux based on differential pH sensitivity [52]. In autophagosomes, both fluorophores emit detectable signals, resulting in yellow puncta in merged images. Once autophagosomes fuse with lysosomes to form autolysosomes, the acidic environment quenches EGFP fluorescence, leaving only red puncta from mCherry. This transition allows discrimination between early and late autophagic structures: an increase in both yellow and red puncta indicates autophagy induction, whereas inhibition at a late step (impaired autophagosome maturation or lysosomal fusion) typically yields a build-up of yellow puncta accompanied by reduced red puncta [53]. Using this principle, CFPAC-1 cells were treated with α-MLT for 24 h, either in the presence or absence of autophagy inhibitors, BafA1 and HCQ. α-MLT treatment led to a clear increase in both yellow and red puncta, indicating autophagy induction. Co-treatment with the inhibitors led to an increased EGFP signal with a concurrent decrease in mCherry signal without an obvious increase in the yellow signal when compared with the monotherapy (Figure 2B). Rapamycin was used as a positive control. Taken together, these data provide evidence of autophagy induction following SLC6A14 blockade.

**Figure 3:**
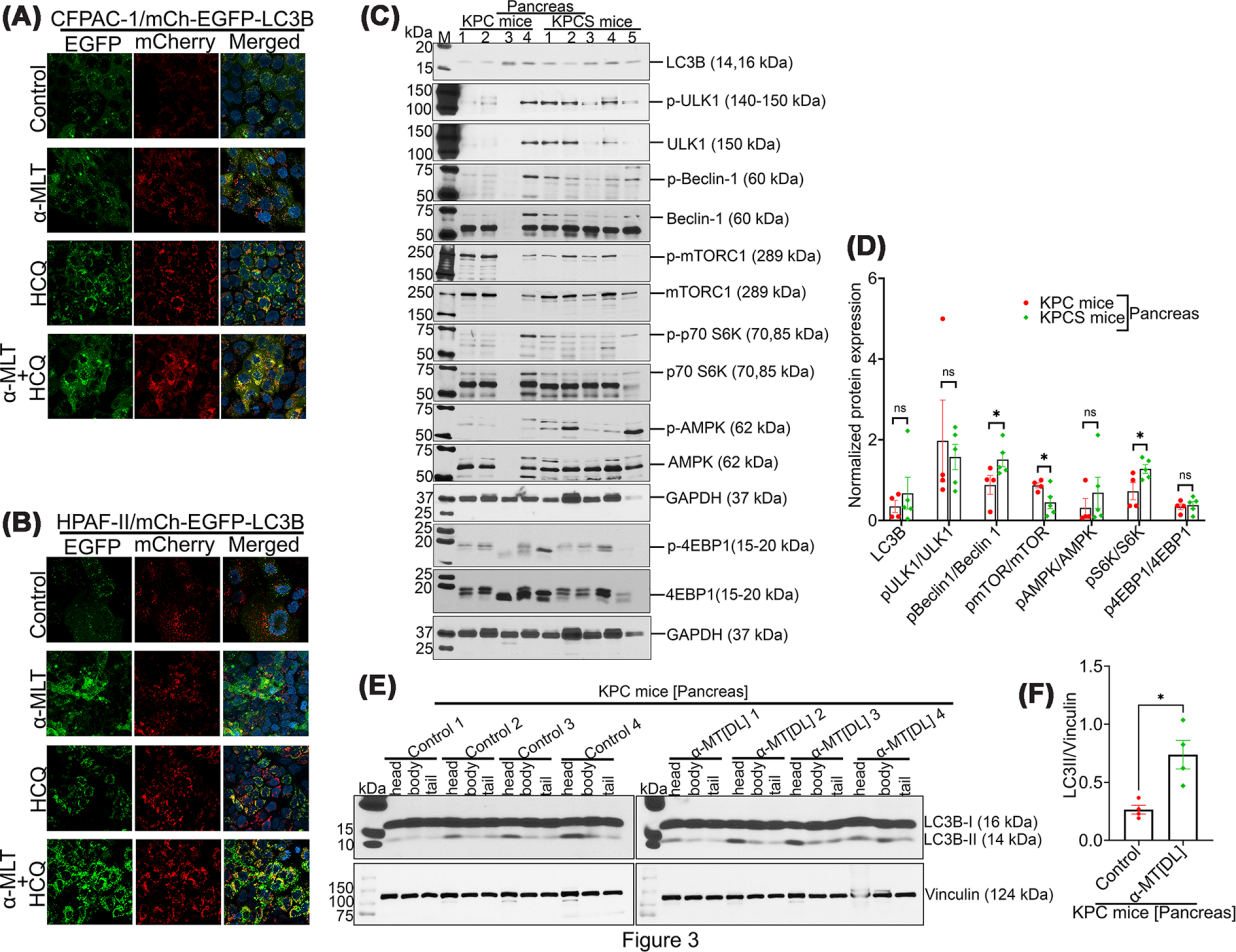
SLC6A14 blockade induces autophagy in PDAC cells and KPC mouse pancreas (**A,B**) Confocal microscopy in CFPAC-1/mCherry-EGFP-LC3B and HPAF-II/mCherry-EGFP-LC3B cells showing EGFP or mCherry signal following treatment with α-MLT, either singly or in combination with HCQ. Image magnification: 100×. (**C**) Western blots showing levels of LC3B, its upstream target proteins, and mTORC1 downstream effector proteins in KPC and KPCS mouse pancreas. GAPDH was used as a loading control. (**D**) Protein bands were quantified by densitometric analysis using ImageJ software. Normalized protein expression was calculated for each target, and the data are presented as the ratio of phosphorylated to the total protein. Data given as mean ± SEM. ns, non-significant; *P <0.05 (**E**) Western blots showing levels of LC3B in KPC/Control and KPC/α-MT[DL]-treated mice pancreas. Vinculin was used as a loading control. (**F**) Densitometric analysis comparing normalized protein expression of LC3B-II between KPC/Control and KPC/α-MT[DL]-treated mouse pancreas. Data given as mean ± SEM. *P <0.05.

Though the autophagic induction was obvious as indicated by the increased EGFP signal following co-treatment with the inhibitors (BafA1 and HCQ), the lack of yellow puncta made us wonder whether cell fixation had any impact on the signals. Therefore, to build on our fixed-cell confocal microscopy, we investigated autophagic dynamics in live cells. Apart from CFPAC-1, we also included HPAF-II cells, which is also a PDAC cell line with high SLC6A14 functionality. CFPAC-1 and HPAF-II cells were treated with α-MLT, either in the presence or absence of HCQ, followed by live imaging. Being an FDA-approved drug, HCQ and not BafA1 was used hereafter as the autophagy inhibitor for all *in vitro* and *in vivo* experiments. Consistent with prior observations, α-MLT treatment led to prominent autophagy induction, evidenced by yellow and red puncta formation. Notably, HCQ co-treatment further enhanced autophagy flux as reflected by the increased yellow puncta and a modest reduction in mCherry-only puncta, indicating lysosomal inhibition and accumulation of autophagosomes (Figure 3A,B). These data support that SLC6A14 blockade with α-MLT induces autophagy in PDAC cells and that HCQ sensitizes cells to flux detection.

To further investigate whether SLC6A14 blockade induces autophagy in an *in vivo* mouse model, we performed Western blotting using pancreases from the KPC (Slc6a14/wildtype; Slc6a14/WT) and KPCS (Slc6a14/knockout; Slc6a14/KO) mice. KPC mice develop full-blown PDAC within 3–4 months. Our rationale was that if SLC6A14 blockade induces autophagy, then deletion of Slc6a14 in the KPC mice should do the same. We tested the levels of LC3B and the phosphorylation status of key autophagy and mTORC1-regulated proteins such as ULK1, Beclin1, mTORC1, pS6 kinase, and 4EBP1. It was interesting to find that the absence of Slc6a14 in the KPC mice had no change in the LC3B levels. However, phosphorylation of Beclin-1 at Ser30 was elevated, consistent with autophagy initiation. Despite suppression of mTORC1, phosphorylation of p70S6K (389/Ser424) increased, suggesting selective modulation of downstream mTORC1 targets (Figure 3C,D). Elevated p-AMPK (Thr172) further supports a metabolic stress response leading to autophagy induction in Slc6a14/KO mice. In contrast, phosphorylation of ULK1 and 4EBP1 remained unchanged. Collectively, these findings indicate that Slc6a14 deletion induces a metabolic stress response that engages components of the autophagy initiation machinery but with heterogenous downstream signaling. This is likely because the KPC and KPCS pancreas samples were collected from aged mice with fully developed tumors, where chronic loss of Slc6a14 may have allowed compensatory rewiring of autophagy and mTORC1 pathways over time, thereby blunting the uniform activation patterns typically observed in acute nutrient-stress models.

Next, we wanted to test whether pharmacological blockade of Slc6a14 in KPC mouse pancreas induces autophagy. For this, Western blotting was performed using pancreas samples (head, body, and tail) collected from eight KPC mice (control = 4; α-MT[DL] treatment = 4) that were treated with and without α-MT[DL] only for two weeks. Although the L-isomer is the blocker of SLC6A14, the racemic mixture α-MT[DL] is more cost-effective and was therefore selected for use in animal studies. Moreover, D isomer of α-MT does not inhibit SLC6A14. Interestingly, short-term treatment with α-MT[DL] resulted in a significant increase in LC3B-II protein levels compared with controls (Figure 3E,F), indicating activation of autophagy under acute SLC6A14 blockade. While additional cohorts and different treatment schedules would further solidify these findings, the current data support the conclusion that short-term pharmacological blockade induces autophagy in KPC mice.

### SLC6A14 blockade induces macropinocytosis in PDAC cell lines and KPC mouse pancreas

**Figure 4:**
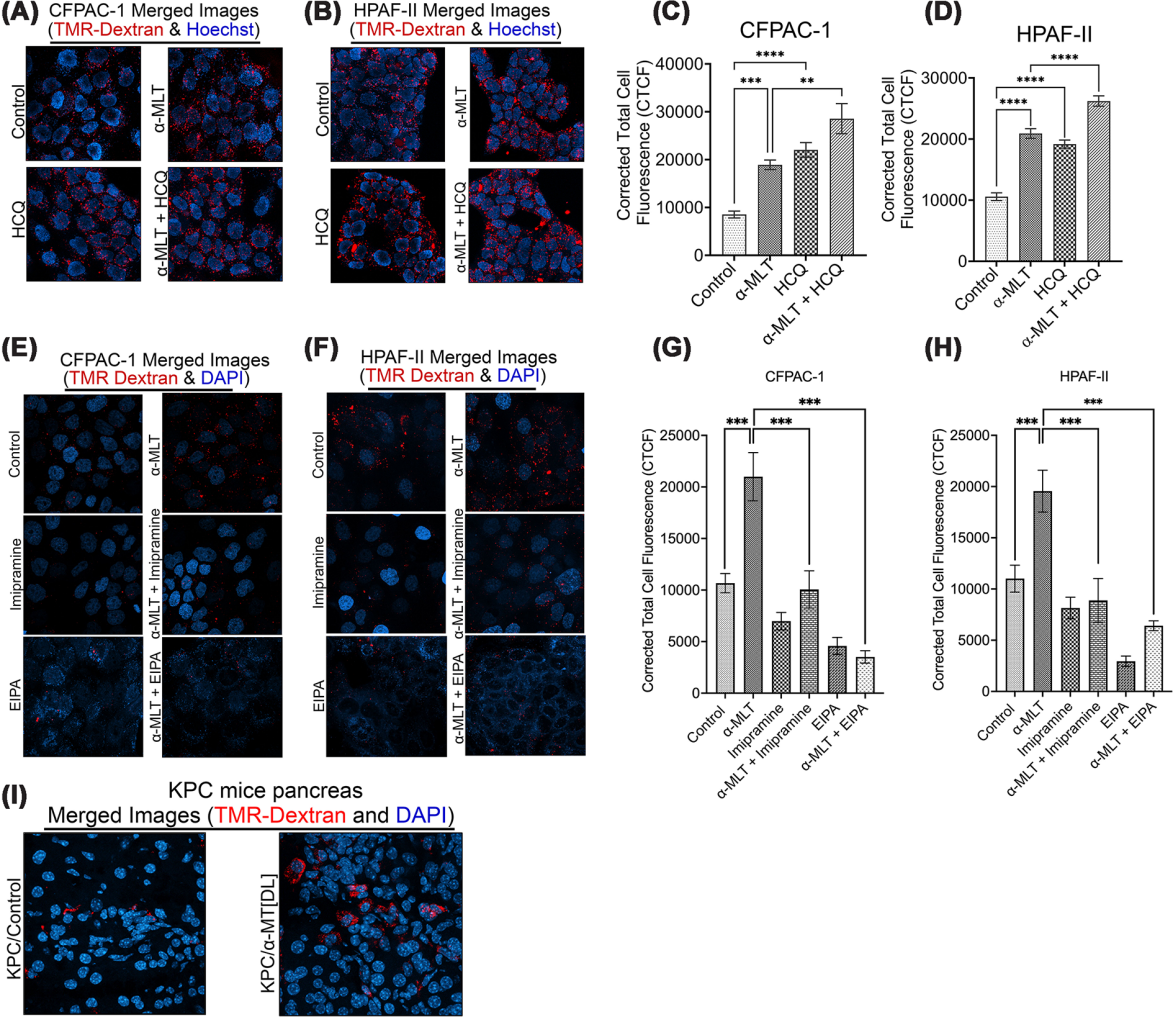
SLC6A14 blockade induces macropinocytosis in PDAC cells and KPC mouse pancreas (**A,B**) Macropinocytosis assay using TMR-dextran as a marker of macropinosomes in CFPAC-1 and HPAF-II cells. Red puncta are macropinosomes. Hoechst, staining blue, identifies nuclei. α-MLT was used as a blocker of SLC6A14. HCQ was used as an inhibitor of macropinocytosis. Image magnification: 100×. (**C,D**) Quantification of the fluorescence signal, measured as the corrected total cell fluorescence using ImageJ software in both CFPAC-1 and HPAF-II cells. Data are given as mean ± SEM. **P <0.01, ***P <0.001, and ****P <0.0001. (**E,F**) Macropinocytosis assay using TMR-dextran as a marker of macropinosomes in CFPAC-1 and HPAF-II cells. Red puncta are macropinosomes. 4',6-diamidino-2-phenylindole (DAPI), staining blue, identifies nuclei. α-MLT was used as a blocker of SLC6A14. Imipramine and EIPA were used as established inhibitors of macropinocytosis, comparing their effects on macropinosome formation both singly and in combination with α-MLT. Image magnification: 100×. (**G,H**) Quantification of the fluorescence signal, measured as the corrected total cell fluorescence using ImageJ software in both CFPAC-1 and HPAF-II cells from panels (E) and (F). Data are given as mean ± SEM. ***P <0.001. (**I**) Representative confocal image showing macropinocytosis signals using TMR-dextran as a marker of macropinosomes in KPC/Control and KPC/α-MT[DL]-treated mouse pancreas. Red puncta are macropinosomes. DAPI, staining blue, identifies nuclei. Image magnification: 100×.

Macropinocytosis is a highly conserved endocytic process by which extracellular fluid and its contents are internalized into cells via large vesicles known as macropinosomes [54,55]. Plasma proteins that extravasate from newly formed and hence leaky tumor blood vessels constitute the major macromolecules in the extracellular milieu for macropinocytosis. Recently, macropinocytosis has emerged as a critical metabolic adaptation process in PDAC that helps it survive the harsh tumor microenvironment. Oncogenic KRAS has been found to drive macropinocytosis in PDAC [56–61]. However, the level of macropinocytosis induced in KRAS mutant cells is found to vary depending on the nutritional status of the cancer cells. Therefore, the macropinocytic drive is not just dependent on the underlying genetic changes but also on the nutritional status [62–65]. Given that SLC6A14 is a broad-selective AAT, its functional inactivation leads to amino acid deprivation and induction of autophagy as a compensatory mechanism of amino acid acquisition. Since macropinocytosis is also a nutrient scavenging mechanism like autophagy, and also given the fact that autophagy cannot continue for prolonged periods of time, we wondered whether macropinocytosis is also induced in conjunction with autophagy to compensate for the amino acid loss. To test this, CFPAC-1 and HPAF-II cells were treated with 2.5 mM α-MLT, with and without HCQ. The cells were also treated with TMR-dextran (70 kDa), used as a fluorescent marker for macropinocytosis, and stained with Hoechst for nuclear visualization. HCQ was used to inhibit macropinocytosis. Though HCQ is a known autophagy inhibitor and is currently under several clinical trials as a combination therapy [66–68], its role in inhibiting macropinocytosis is not well established. Nonetheless, since HCQ inhibits autophagy by inhibiting autolysosomes, conceptually, HCQ should also inhibit macropinolysosomes, since the endpoint of both these processes is lysosomal degradation of their substrates. Live-cell confocal imaging revealed basal macropinocytic activity indicated by the red TMR dextran puncta, which is expected from mutant KRAS cells. Interestingly, treatment with α-MLT significantly enhanced macropinosome formation in both CFPAC-1 and HPAF-II cells as indicated by the increased TMR dextran signal and the elevated corrected total cell fluorescence (Figure 4A–D). Interestingly, HCQ treatment further amplified the TMR-dextran accumulation, suggesting that inhibition of lysosomal fusion of the macropinosomes prevented degradation of the macropinocytic cargo, leading to increased flux. Since HCQ is not a classical macropinocytosis inhibitor, its effects reflect impaired processing of macropinosomes rather than inhibition of macropinocytosis initiation. To validate that the α-MLT-induced increase in TMR-dextran puncta reflects bonafide macropinocytosis, we treated CFPAC-1 and HPAF-II cells with α-MLT in the presence or absence of 5-(N-Ethyl-N-isopropyl)amiloride (EIPA) and imipramine, both of which are known macropinocytosis inhibitors. Both inhibitors markedly reduced basal macropinocytosis as well as α-MLT-induced increase in TMR-dextran puncta, confirming that SLC6A14 blockade stimulates macropinocytosis (Figure 4E,F). This is also indicated by the reduced corrected total cell fluorescence (Figure 4G,H).

We further extrapolated these data using pancreases collected from four KPC mice that were treated with and without α-MT[DL] for one week. The increased TMR-dextran puncta in the KPC/α-MT[DL]-treated mice as opposed to the KPC/control counterparts indicated that Slc6a14 blockade does induce macropinocytosis in the *in vivo* setting as well (Figure 4I). Nevertheless, expanding the KPC cohorts would strengthen the statistical significance. Together, these findings demonstrate that SLC6A14 blockade also induces macropinocytosis, validating our hypothesis that macropinocytosis is up-regulated in response to amino acid deprivation.

### Combination of α-MLT and HCQ synergizes to reduce proliferation and clonogenic ability in PDAC cells

**Figure 5:**
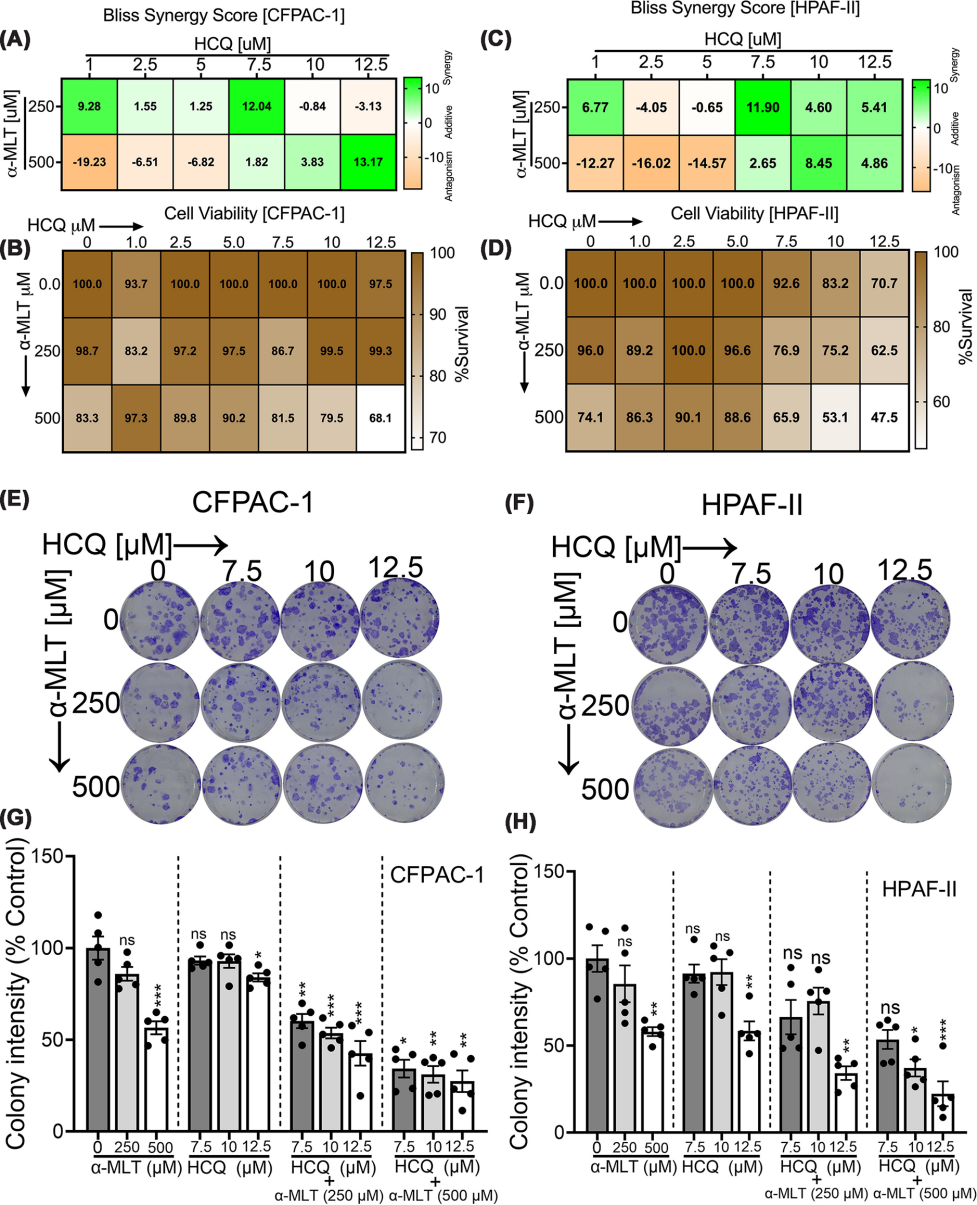
Combination of α-MLT and HCQ synergizes to reduce proliferation and clonogenic ability in PDAC cell lines Bliss synergy score and cell viability assay in CFPAC-1 (**A,B**) and HPAF-II (**C,D**) cells showing synergistic effects in the combination studies with HCQ and α-MLT as opposed to monotherapy. (**E,F**) Colony formation assay in CFPAC-1 and HPAF-II PDAC cell lines treated with α-MLT and HCQ, either singly or in combination. (**G,H**) Colony quantification in CFPAC-1 and HPAF-II PDAC cell lines treated with α-MLT and HCQ, either singly or in combination. Data are given as mean ± SEM. ns, non-significant; *P <0.05; **P <0.01; and ***P <0.001.

Having established that blockade of SLC6A14 induces autophagy as well as macropinocytosis in PDAC cells and KPC mice, we then hypothesized that the combination of α-MLT and HCQ will have better efficacy in terms of cell proliferation and growth *in vitro*. To test this, MTT cell viability assay was performed in both CFPAC-1 and HPAF-II cells using two different concentrations of α-MLT and multiple concentrations of HCQ, either singly or in combination. The results from these experiments showed that the combination of the two drugs was superior in lowering the proliferation capacity of the cells. However, HPAF-II cells exhibited a better outcome than CFPAC-1 cells (Figure 5B,D). Using the MTT data, Bliss synergy analysis was performed, which indicated that the observed outcome in the combination group was synergistic in both the cell lines tested (Figure 5A,C). Additionally, a colony formation assay was also performed in both CFPAC-1 and HFAF-II cell lines using two different concentrations of α-MLT and multiple concentrations of HCQ. The data clearly indicated that while α-MLT by itself at 250 and 500 μM was able to lower the clonogenic ability in CFPAC-1 and HPAF-II cells, co-treatment with HCQ (7.5, 10, and 12.5 μM) resulted in an even greater reduction in colony formation in both the cell lines (Figure 5E,F) as validated by quantification analyses (Figure 5G,H). Collectively, these findings demonstrate that α-MLT alone has notable antiproliferative effects and that these effects are further enhanced through combination with HCQ, thus underscoring the therapeutic potential of the dual targeting strategy in PDAC.

### Combination of α-MT[DL] and HCQ did not lead to a better therapeutic outcome as opposed to monotherapy in the prevention model of xenograft study

**Figure 6:**
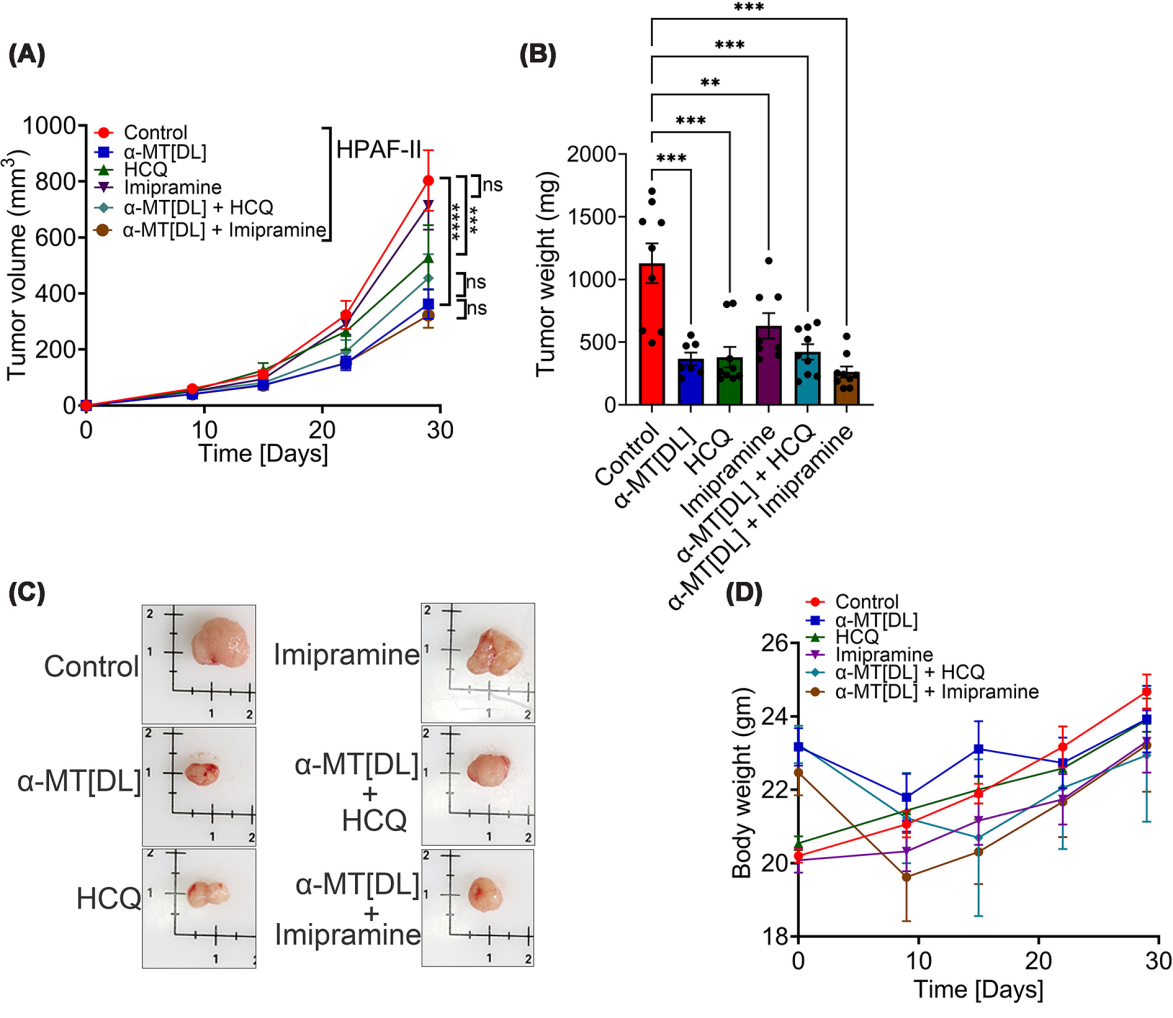
Combination of α-MT[DL] and HCQ did not lead to a better therapeutic outcome as opposed to monotherapy in the prevention model of xenograft study (**A,B**) Tumor volume and tumor weight in athymic nude mice subcutaneously xenografted with HPAF-II cells and treated with α-MT[DL], HCQ, and imipramine, either singly or in combination. (**C**) Representative tumor samples extracted from six different groups at the end of the study. (**D**) Body weight of the mice from different groups of the xenograft study. Data are given as mean ± SEM. ns, non-significant, **P <0.01, ***P <0.001, ****P <0.0001.

After demonstrating that the combination of α-MLT and HCQ was superior as opposed to α-MLT by itself in reducing the proliferation capacity of the PDAC cells in the *in vitro* assays, we then wanted to test whether similar results could be obtained in an *in vivo* mouse study. We opted for a prevention model first to test whether early intervention can alter the tumor growth and thereby have a different therapeutic outcome. For this, HPAF-II cells were subcutaneously xenografted in female athymic nude mice, and post-injection, mice were divided into six groups (control, α-MT[DL], HCQ, imipramine, α-MT[DL] + HCQ, and α-MT[DL] + imipramine), each consisting of 5 mice/group. The rationale for choosing HPAF-II over CFPAC-1 for the xenograft study was due to the reliable tumor growth kinetics in the athymic nude mice with this cell line. The drugs were provided in drinking water at 2 mg/ml of α-MLT and 0.3 mg/ml of both HCQ as well as imipramine, and the treatment started on the same day the cancer cells were injected into the mice. Due to the cost, α-MT[DL] racemic mix instead of α-MLT was used for all animal-related works. D-isomer of α-MT does not block SLC6A14, and therefore the observed outcome in tumor attenuation as a result of SLC6A14 blockade is mediated by α-MLT. Imipramine was also included in the study since it is an FDA-approved tricyclic antidepressant and a highly potent inhibitor of macropinocytosis [69]. Since SLC6A14 blockade induced autophagy and macropinocytosis and since the combination drugs worked better in the *in vitro* studies, we predicted a superior outcome in terms of tumor attenuation in the combination therapy as opposed to monotherapy. Contrary to our expectation, α-MT[DL] by itself was able to significantly reduce the tumor volume by more than 50% when compared with the control. More importantly, combining α-MT[DL] with either HCQ or imipramine did not improve the result any further, as evident from the tumor volume and tumor weight (Figure 6A–C). HCQ and imipramine by themselves also reduced the tumor volume by more than 50% and 40%, respectively, when compared with control. With regard to body weight, α-MT[DL] by itself and its combination with HCQ and imipramine showed a significant decrease in the beginning of the experiment; however, the animals regained their weight during the course of the experiment, which remained stable until the end (Figure 6D). Taken together, the data show that in the prevention model of the xenograft study, monotherapy was as good as combination therapy in attenuating tumor growth in athymic nude mice.

### Combination of α-MT[DL] and HCQ led to a better therapeutic outcome as opposed to monotherapy in the treatment model of xenograft study

**Figure 7:**
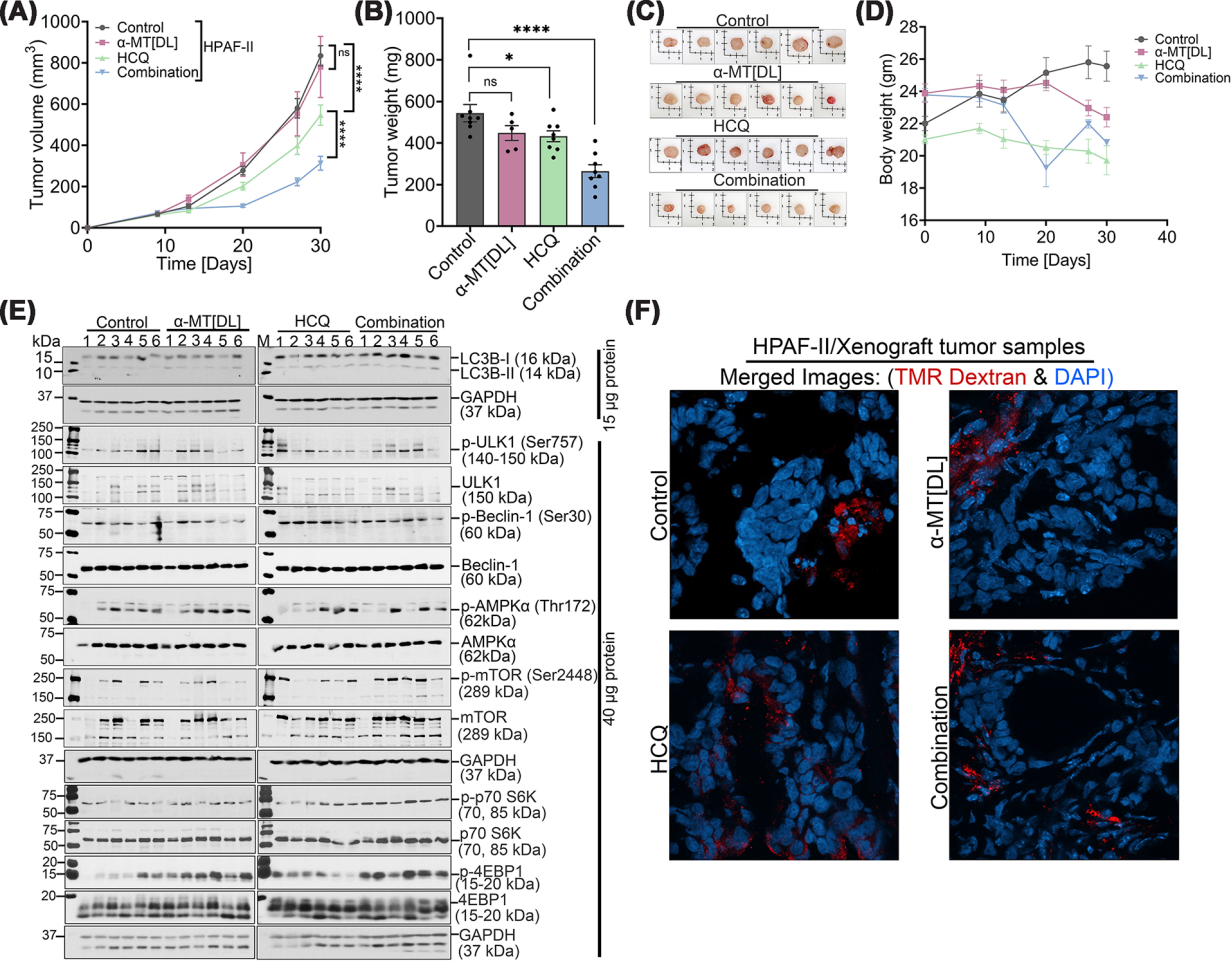
Combination of α-MT[DL] and HCQ led to a better therapeutic outcome as opposed to monotherapy in the treatment model of xenograft study (**A,B**) Tumor volume and tumor weight in athymic nude mice subcutaneously xenografted with HPAF-II cells and treated with α-MT[DL] and HCQ, either singly or in combination. (**C**) Representative tumor samples extracted from the control and treatment groups at the end of the study. (**D**) Body weight of the mice from the control and treatment groups. (**E**) Western blot showing levels of LC3B, its upstream target proteins, and mTORC1 downstream effector proteins in the tumor samples from the control and treatment groups. GAPDH was used as the loading control. (**F**) Representative images showing the macropinocytosis uptake assay using TMR-dextran as a marker of macropinosomes in control and the treatment groups. Red puncta are macropinosomes. DAPI, staining blue, identifies nuclei. Image magnification: 100×. Data are given as mean ± SEM. ns, non-significant; *P <0.01; ****P <0.0001.

Since the combination of α-MT[DL] and HCQ did not outperform monotherapy in the prevention model, we hypothesized that early administration prior to proper xenograft establishment may have contributed to such an unexpected outcome by prioritizing SLC6A14 blockade over autophagy/macropinocytosis inhibition as the primary cause of attenuation of tumor growth. This could have curtailed nutrient uptake, disrupted initial tumor growth, and diminished the potential efficacy of combination therapy. To test this, a follow-up subcutaneous xenograft was performed in female athymic nude mice by altering the timeline of the drug treatment. The animals were divided into 4 groups (*n* = 5 per group: control, α-MT[DL], HCQ, and combination). All groups exhibited comparable tumor sizes at baseline (100 mm^3^), and the treatment began only after tumors reached this threshold. Drug concentrations mimicked those used in the prevention model. Interestingly, the combination regimen drastically reduced the tumor volume when compared with either monotherapy, as depicted by the tumor volume, tumor weight, and the representative tumor images (Figure 7A–C). Given the slight body weight reduction observed previously in α-MT[DL] and the combination groups under the prevention model, supplemental DietGel cups were provided in addition to the regular chow to all the entire groups. Though some mild weight fluctuations persisted in the combination, these were not life-threatening (Figure 7D). Overall, the data demonstrated that the combination of α-MT[DL] and HCQ is better than α-MT[DL] by itself in attenuating tumor growth in the treatment model.

After confirming a better therapeutic outcome in the combination group as opposed to the monotherapy, we wanted to test whether the observed effect was due to inhibition of autophagy and/or macropinocytosis in addition to blockade of SLC6A14. Western blotting was performed using tumor samples (*n* = 6 per group) from all the groups and probed for LC3B and the phosphorylation status of key autophagy and mTORC1-regulated proteins such as ULK1, Beclin1, mTORC1, pS6 kinase, and 4EBP1. While phosphorylated ULK1, Beclin-1, AMPKα, p70S6K, and mTORC1 remained largely unchanged across all treatment groups based on the densitometric quantification and composite data from six tumors, it is still true that LC3B-I, instead of LC3B-II, as well as p-mTORC1, was up-regulated in at least a few subsets of tumor samples from the combination group when compared with either of the monotherapy or the control groups (Figure 7E and Supplementary Figure S1). Given the reciprocal relationship between autophagy and mTORC1 signaling, these data suggest that activated mTORC1 in the combination group may have inhibited the autophagic precursor activity, thereby inhibiting autophagosome formation and downstream autophagy.

To assess the macropinocytosis status in these mice, TMR-dextran was intratumorally injected as the fluorescent tracer dye in the live athymic nude mice, and the tumors were extracted and frozen in OCT an hour post-injection. As HCQ impedes the fusion of macropinosomes with lysosomes, accumulation of TMR-dextran puncta serves as a marker for macropinocytosis blockade. Though the KPC mice, upon a short-term exposure to α-MT[DL], induced macropinocytosis, we have to keep in mind that in the xenograft study, the animals were continuously exposed to the drugs and therefore might have a totally different outcome. The data clearly showed an increase in TMR-dextran puncta in α-MT[DL]-treated and HCQ-treated groups; however, the puncta size and intensity were higher in the combination, indicating macropinocytosis inhibition (Figure 7F). Collectively, the data indicate that α-MT[DL] in combination with HCQ is better than α-MT[DL] monotherapy in attenuating tumor growth. We also observed that while prolonged treatment produced heterogeneous changes in autophagy-related markers, the therapeutic advantage of the combination therapy is consistent with broader disruption of metabolic-stress response pathways in response to SLC6A14 blockade.

## Discussion

PDAC is highly aggressive and up-regulates SLC6A14 to meet the high amino acid demand for their growth and metabolism. We have already published using athymic nude mice and KPC spontaneous mouse model of PDAC that blockade of SLC6A14 attenuates tumor growth as well as improves overall survival. Mechanistically, we have shown that SLC6A14 blockade leads to amino acid deprivation and inhibition of mTORC1 signaling pathway, ultimately leading to PDAC attenuation [12,35]. While several studies have reported induction of autophagy and macropinocytosis in response to amino acid deprivation [16], it remains unclear whether SLC6A14 blockade triggers these responses in PDAC. This question is critical, as the induction of autophagy/macropinocytosis could potentially undermine the full anticancer efficacy of SLC6A14 blockade by enabling tumor cells to compensate for nutrient loss. Therefore, the present study was designed to investigate whether SLC6A14 blockade induces autophagy and/or macropinocytosis in PDAC cells and, if true, whether a combination strategy using α-MLT, a selective blocker of SLC6A14, together with HCQ, an inhibitor of autophagy and macropinocytosis, would yield superior antitumor efficacy compared with α-MLT monotherapy.

Here, we demonstrate for the first time that SLC6A14 blockade induces both autophagy and macropinocytosis in PDAC cell lines and, in short-term, *in vivo* experiments in KPC mice. SLC6A14 is a sodium-coupled, broad-selective AAT with the ability to transport 18 of the 20 amino acids in a unidirectional and concentrative manner [13–15,70–72]. Therefore, PDAC cells reap the benefit from SLC6A14 up-regulation. Though it is known that autophagy and macropinocytosis are observed at a higher basal level due to the hypovascularized nature of PDAC [16–18], loss of amino acids can further accentuate these effects. While cancer cells also require sugars and lipids, amino acids in particular are critical for cancer growth and, as intermediates, connect glucose, lipid, and nucleotide metabolism [7,71–73]. Glutamine is an excellent substrate for SLC6A14; this amino acid supports glutaminolysis in cancer cells. This pathway not only leads to energy production but also generates lactic acid, which is now recognized as a tumor-promoting oncometabolite [74]. Glutamine is also involved in lipid synthesis in cancer cells via a selective metabolic pathway known as reductive carboxylation [75]. Serine, glycine, and methionine are also good substrates for SLC6A14; all these three amino acids are the drivers of one-carbon metabolism involved in nucleotide synthesis and epigenetic regulation via lysine/arginine methylation and DNA methylation [13]. Of course, since SLC6A14 is capable of transporting 18 of the 20 amino acids, its involvement in promoting protein synthesis is quite obvious. Though autophagy and macropinocytosis are separate strategies, they are also very collaborative in a general sense. While autophagy can prolong the cell viability by consuming intracellular macromolecules, this process can lead to cell shrinkage and loss of biomass, which would eventually become detrimental for the cancer cell proliferation. Therefore, in a very concerted manner, cancer cells induce macropinocytosis following autophagy induction so that they can maintain the viability as well as continue with their upstream signaling [16]. The major substrates for macropinocytosis are the plasma proteins that leak out of newly formed blood vessels in tumors. Thus, given the importance of amino acids in cancer cell proliferation and the role SLC6A14 plays in fulfilling this requirement, it is logical to think why PDAC cells up-regulate both autophagy and macropinocytosis in response to its blockade.

Since autophagy and macropinocytosis are adaptive strategies used by cells to scavenge and recycle essential nutrients for survival, using combination agents to block this aspect of PDAC metabolism has therapeutic potential. Targeting SLC6A14 and both the potential escape mechanisms for SLC6A14 inhibition concurrently provides an innovative approach. Though literature evidence shows dual inhibition of autophagy and macropinocytosis as a strategy to treat PDAC, our approach to metabolically collapse the PDAC cells by blocking both the cause (SLC6A14) and the effect of its inhibition (autophagy and macropinocytosis) is the first of its kind. Our study shows a synergistic effect with the combination of α-MLT and HCQ, as evidenced by the reduced cell viability and the clonogenic ability of the PDAC cells. This suggests that dual inhibition of nutrient uptake and adaptive stress responses enhance cytotoxicity. Our results align with previous studies showing that dual inhibition sensitizes PDAC cells to metabolic stress and chemotherapy [76]. Additionally, HCQ is a known autophagy inhibitor and is currently under several clinical trials as a combination therapy [66,67]; however, its role in inhibiting macropinocytosis is not well established. Nonetheless, since HCQ inhibits autophagy by inhibiting autolysosomes, conceptually, HCQ should also inhibit macropinolysosomes, since the endpoint of both these processes is lysosomal degradation of their substrates. Our *in vitro* results validate this and further support the rationale for targeting both SLC6A14 and autophagy/macropinocytosis.

Furthermore, we also show a context-dependent therapeutic response in the mouse subcutaneous xenograft study wherein α-MT[DL] monotherapy was as good as the combination therapy in attenuating the tumor growth in a preventive model. On the contrary, combination therapy clearly outperformed monotherapy in a treatment model. This suggests that targeting SLC6A14 alone may be sufficient to prevent tumor initiation in the context of a preneoplastic disease; however, combination therapy is the key to treating more advanced tumors that may rely more heavily on autophagy and macropinocytosis for survival and progression. Studies have shown that late-stage PDAC exhibits heightened metabolic flexibility and reliance on scavenging pathways [77–79]. Interestingly, chronic treatment in the xenograft study showed compensatory signaling patterns that differed from the acute responses observed *in vitro* and in short-term KPC studies. Canonical autophagy markers like the phospho-ULK1, Beclin 1, and AMPK1 remained unchanged across treatment groups; p-mTORC1(S2448) and LC3B-I showed modest increases in the combination group with no change in LC3B-II. This suggests that chronic nutrient stress in the tumor microenvironment triggers adaptive rewiring that obscures static autophagy markers. Rather than indicating absence of autophagy stress, these patterns likely reflect impaired autophagic flux and compensatory reactivation of mTORC1. Thus, the therapeutic benefit of the combination therapy is best explained by disruption of nutrient acquisition and metabolic resilience rather than by simple inhibition of autophagy initiation.

The dosing regimens for the drugs were selected based on previous studies. α-MLT is currently used as a PET probe for serotonergic neurons in brain [80]. It may also have use in the treatment of depression [81]. These uses of α-MLT are based on its conversion *in vivo* into α-methylserotonin, which can act as a serotonin substitute. Because of these potential therapeutic uses, studies have already been done on the toxicology and pharmacokinetics of α-MLT in animals and humans [80,81]. In mice and rats, there were no serious adverse effects at an oral dose of 150 mg/kg [82,83]. In fact, there were positive effects due to the drug in social behavior such as self-grooming and burrowing. HCQ is widely used to inhibit autophagy, exhibits high oral bioavailability (∼70%–80%) and extensive tissue accumulation, resulting in sustained intracellular concentrations and prolonged half-life [84]. Similarly, imipramine, a lipophilic tricyclic antidepressant, is rapidly absorbed and widely distributed in tissues, allowing effective intracellular concentrations that have been reported to inhibit macropinocytosis [85]. Therefore, the concentrations used in the present study are consistent with previously reported *in vivo* dosing regimens that help achieve pharmacologically relevant systemic exposure and thereby target tumor tissues.

Taken together, our findings identify SLC6A14 as a metabolic target in PDAC and show that its blockade induces autophagy and macropinocytosis. Dual inhibition of SLC6A14 and the lysosome-dependent nutrient scavenging pathways creates a metabolic trap that PDAC cells cannot escape, culminating in tumor attenuation. This strategy represents a promising therapeutic avenue that warrants further evaluation in the PDAC model.

## Conclusions

**Figure 8:**
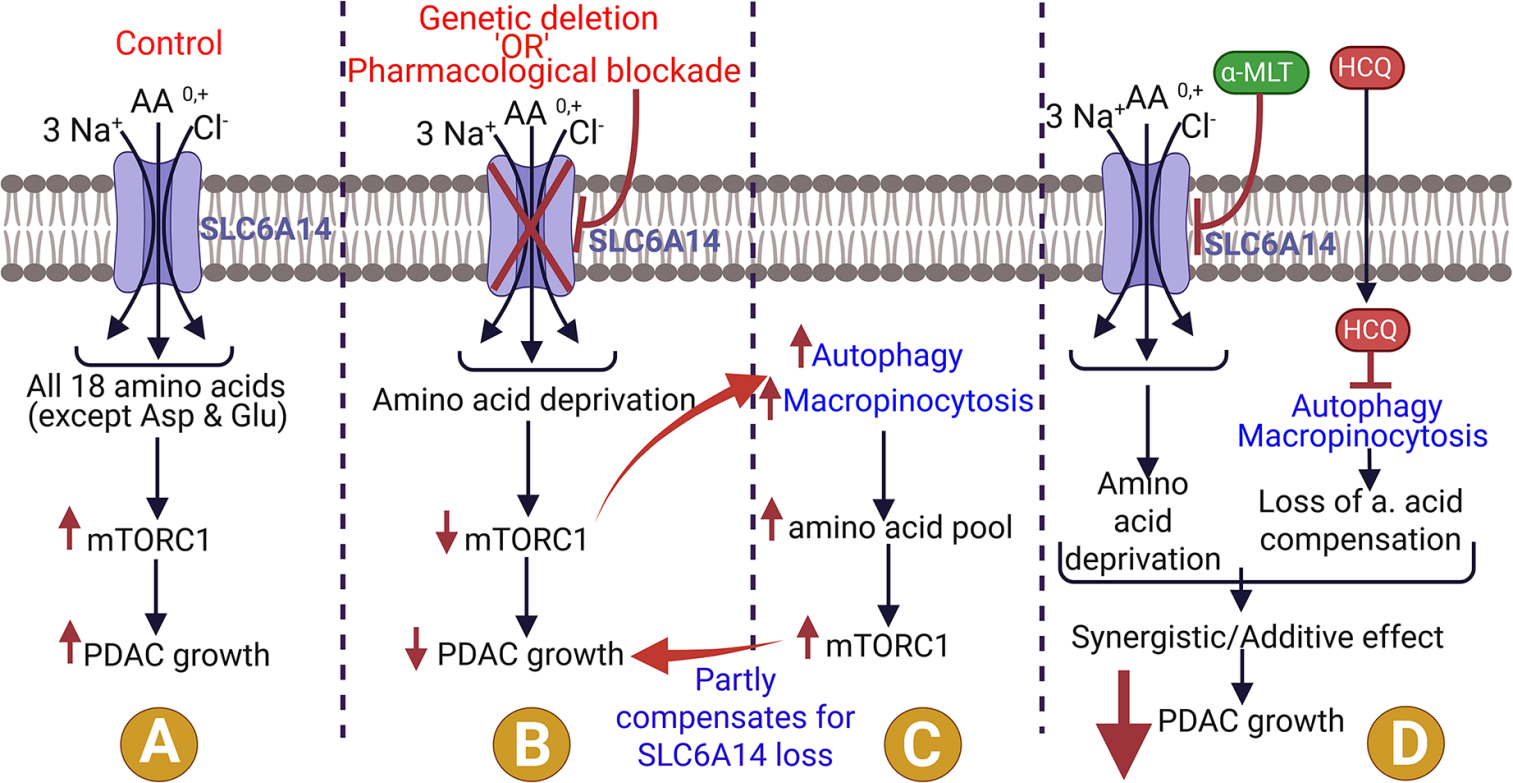
Dual targeting of SLC6A14 and compensatory nutrient acquisition pathways suppresses PDAC growth (**A**) SLC6A14 promotes PDAC by translocating amino acids into tumor cells, thereby activating mTORC1 signaling pathway. (**B**) Genetic deletion or pharmacological blockade of SLC6A14 leads to amino acid deprivation, mTORC1 inhibition, and PDAC attenuation (published work). (**C**) Here, we show induction of autophagy and macropinocytosis in response to SLC6A14 blockade that replenishes intracellular amino acid pools, which potentially reactivates mTORC1, and thereby partly compensates for SLC6A14 loss. (**D**) Co-inhibition of both arms, SLC6A14 (via α-MLT) and autophagy and macropinocytosis (via HCQ), yields a synergistic/additive therapeutic effect, suppressing PDAC growth in xenograft mouse models.

Based on our previously published work that SLC6A14 promotes PDAC by activating mTORC1, we show that inactivation of SLC6A14, either through genetic deletion or pharmacological blockade with α-MLT, leads to amino acid deprivation, mTORC1 inhibition, and PDAC attenuation. Extending these findings, we now demonstrate that SLC6A14 blockade induces autophagy and macropinocytosis, which may replenish intracellular amino acid pools and reactivate mTORC1, thereby partially compensating for the loss of SLC6A14. To overcome this adaptive resistance, we employed a dual therapeutic strategy targeting both SLC6A14 (α-MLT) and autophagy and macropinocytosis (HCQ) and showed that combination therapy yielded a better therapeutic outcome in PDAC models as opposed to targeting SLC6A14 alone (Figure 8).

## Materials and methods

### Cell lines and culture conditions

CFPAC-1 and HPAF-II were obtained from ATCC and were used within 10–20 passages. For characterization of these cell lines, ATCC has performed morphological, cytogenetic, and DNA profile analyses. The cell lines were maintained in the respective media recommended by the ATCC (Iscove’s DMEM for CFPAC-1 and Eagle's Minimum Essential Medium for HPAF-II) supplemented with 10% FBS. Moreover, the media contained 100 U/ml penicillin and 100 U/ml streptomycin. The culture media for the above cell lines were purchased from Corning (Manassas, VA, U.S.A.), whereas FBS was from Gibco (U.S.A., Cat# 26140079), and plastic culture dishes for cell culture were obtained from Corning Life Sciences (Manassas, VA, U.S.A.). Cells were cultured at 37°C in a humidified atmosphere containing 5% CO_2_. The cell lines have been routinely tested for mycoplasma contamination using the Mycoplasma PCR Detection Kit obtained from Applied Biological Materials (abm Cat# G238). Cells were discarded immediately if tested positive for mycoplasma.

### Chemical reagents and antibodies

α-MLT (L-isoform) was purchased from 3B Scientific Corporation Limited (Wuhan, China). α-MT (DL racemic mixture) was purchased from BACHEM (Cat# 4008387). Rapamycin was from Santa Cruz Biotechnology (Cat# sc-3504A). Bafilomycin A1 was from Millipore Sigma (Cat# SML1661). Hydroxychloroquine (HCQ) sulfate was obtained from SelleckChem (Cat# S4430). Imipramine hydrochloride (Cat# I7379) and EIPA (Cat# A3085) were from Sigma. 70 kDa TMR-Dextran (Cat# D1818) and 10 kDa TMR-Dextran (Cat# D1817) were purchased from ThermoFisher Scientific. Bicinchoninic acid protein assay reagent (Cat# 23225), RIPA buffer (Cat# 89901), and Protease and Phosphatase Inhibitor Cocktail (Halt) (Cat# 78442) were from ThermoFisher Scientific. MTT reagent (Cat# 475989) was from Sigma. Crystal violet was from Remel Inc. (Lenexa, KS, U.S.A.). The GAPDH mouse monoclonal (Cat# sc-47724) primary antibody was from Santa Cruz Biotechnology (Dallas, Texas, U.S.A.). Other primary antibodies like LC3B rabbit polyclonal (Cat# 2775), phospho-Beclin-1 (Ser30) rabbit polyclonal (Cat# 54101), Beclin-1 rabbit monoclonal (Cat# 3495), phospho-ULK-1 (Ser757) rabbit monoclonal (Cat# 14202), ULK-1 rabbit monoclonal (Cat# 8054), phospho-AMP-kinase (Thr172) rabbit monoclonal (Cat# 2535), AMP-kinase rabbit polyclonal (Cat# 2532), phospho-mTOR (Ser2448) rabbit monoclonal (Cat# 5536), mTOR rabbit monoclonal (Cat# 2983), phospho-p70 S6 kinase (Thr389) rabbit monoclonal (Cat# 9234), p70 S6 kinase rabbit monoclonal (Cat# 34475), phospho-4E-BP1 (Ser65) rabbit polyclonal (Cat# 9451), 4E-BP1 rabbit monoclonal (Cat# 9644), and Vinculin rabbit monoclonal (Cat# 13901) were procured from Cell Signaling Technology (Danvers, MA, U.S.A.). Secondary antibodies like goat anti-rabbit IgG (Cat# 170-6515) and goat anti-mouse IgG (170-6516) were procured from Bio-Rad Laboratories (Hercules, CA, U.S.A.).

### Retroviral expression plasmids, transfection, and transduction

Retroviral expression vector with a pBABE-puro backbone was used to overexpress mCherry-EGFP-LC3B (Addgene plasmid #22418) in CFPAC-1 and HPAF-II cell lines. pCL-10A1 retrovirus packaging vector was purchased from Novus Biologicals (Cat# NBP2-29542). Lipofectamine 3000 (Cat# L3000015) was purchased from ThermoFisher Scientific. Briefly, for plasmid transfection, 7.5 μg of packaging DNA and 7.5 μg of expression plasmid DNA were combined with Opti-MEM and transfected with Lipofectamine 3000, according to the manufacturer’s protocol. The transfection mixture was incubated at room temperature for 20 min. After that, 10 × 10^6^ of HEK293FT cells, suspension, and transfection mixture were mixed gently and plated in a fresh culture dish and placed in a humidified incubator at 37°C. The following day, the HEK293FT cells’ medium was changed to a fresh one supplemented with 20% FBS. After 48 h, the viral particles in media were harvested, filtered using a 0.45 μm syringe filter, and then applied to target cells. For retroviral transduction, CFPAC-1 and HPAF-II cells were plated and grown to 60%–70% confluency. Thereafter, media containing viral particles were added to target cells along with an 8 μg/ml final concentration of polybrene. The viral medium was replaced with complete medium after 24 h, and cells were maintained in complete medium for 24 h. After 24 h, cells underwent selection for 14 days with 1 μg/ml puromycin (CFPAC-1) and 0.5 μg/ml puromycin (HPAF-II). Thereafter, all transduced cells were maintained in complete medium with puromycin. Overexpression of mCherry-EGFP-LC3B in both cell lines was confirmed using confocal microscopy after detecting mCherry and EGFP fluorescence signals compared with negative control parental cells.

### *In vitro* macropinocytosis assay

*In vitro* macropinocytosis assay was conducted as described previously [23–26]. High molecular weight 70 kDa TMR (tetramethylrhodamine)-dextran was used to monitor macropinocytosis status in CFPAC-1 and HPAF-II cells under various treatment conditions. Briefly, cells were plated onto coverslips (25 mm diameter, 1.5 thickness), placed in a six-well plate at a density of 2 × 10^5^ cells/well (CFPAC-1) and 3 × 10^5^ cells/well (HPAF-II), and cultured at 37°C until reaching ∼70% confluency. The cells were washed three times with a buffer consisting of 140 mM NaCl, 5.4 mM KCl, 1.8 mM CaCl_2_, 0.8 mM MgSO_4_, 5 mM glucose, and 25 mM HEPES (pH 7.5). The cells were then incubated with TMR-dextran (100 μg/ml) along with various treatment conditions (control, α-MLT 2.5 mM, HCQ 20 μM, α-MLT + HCQ) in the same buffer at 37°C for 1 h. Following this, the cells were washed three times using the same assay buffer and incubated for 5 min with 1 μg/ml Hoechst 33342 before imaging.

Macropinocytosis was also performed using additional macropinocytosis inhibitors like EIPA and imipramine, both singly and in combination with α-MLT. Cells were plated onto coverslips and cultured at 37°C, allowing them to reach around ∼70% confluency before the start of the experiment. Prior to treatment, the cells were washed three times with the assay buffer described above. Cells were then incubated with TMR-dextran (100 μg/ml) under the indicated treatment conditions (control, α-MLT 2.5 mM, imipramine 5 μM, α-MLT + imipramine) in the same buffer at 37°C for 1 h. For EIPA treatment, cells were incubated with 10 μM EIPA for 30 min. Following incubation, the cells were washed three times with the assay buffer and fixed in 2% paraformaldehyde for 10 min. Following three PBS washes, the cells were mounted using Prolong diamond with DAPI and allowed to cure overnight before imaging.

Cells were imaged using laser scanning confocal microscopy (Nikon T1-E) with a 100× objective and analyzed with NIS software. Seven regions of interest with 15–20 cells were imaged randomly. The images represent a maximum projection intensity derived from the Z-stack.

The fluorescence quantification was performed by measuring the corrected total cell fluorescence (CTCF) using Image J and the following formula:

CTCF = (integrated density) - (area of cell of interest) × (mean fluorescence of background).

An outline of each cell was drawn to measure the integrated density, area of the cells of interest, and mean fluorescence of the adjacent background around the cell of interest.

### *In vivo* macropinocytosis assay

*In vivo* macropinocytosis assay was performed based on a standard protocol [26]. Briefly, pancreas samples from KPC mice and tumor samples from HPAF-II/xenograft studies were used. TMR-dextran (10 kDa) was used. For the KPC spontaneous mouse model of PDAC, 1 mg of TMR-dextran dissolved in PBS was injected intravenously. After 30 min, the pancreas was harvested and embedded in OCT compound and frozen. Frozen sections were mounted using Prolong Diamond with DAPI as a nuclear marker. The data were obtained from two control KPC mice compared with two KPC mice treated with α-MT. For subcutaneous xenograft athymic nude mice, TMR-dextran dissolved in PBS was injected intratumorally. 1 mg of TMR-dextran was injected for a 500 mm^3^ tumor, which was used as a standard. The amount of dextran injected was adjusted based on the volume for every tumor. After 60 min, the tumors were harvested and embedded in OCT compound in cryomold and frozen. Frozen sections were mounted using Prolong Diamond with DAPI as a nuclear marker. Two tumors were analyzed per group (control versus α-MT versus HCQ versus combination). Sections were imaged using laser scanning confocal microscopy (Nikon T1-E) with a 100× objective. Five random regions of interest were imaged for each sample. The images represent a maximum projection intensity derived from the Z-stack.

### Western blot analysis

Western blotting was performed as previously described [11,12]. In short, cells were grown to 70%–80% confluency, and following treatments in reduced serum conditions with α-MLT, BafA1, HCQ, and respective combinations, whole-cell lysates were prepared in RIPA lysis buffer (Pierce) with a protease and phosphatase inhibitor cocktail (Halt). Pancreatic tissue samples from KPC mice and KPCS mice and subcutaneous xenograft tumors were homogenized, and tissue lysates were prepared in RIPA lysis buffer (Pierce) with a protease and phosphatase inhibitor cocktail (Halt). For both whole-cell lysates and tissue samples, equal amounts of protein were loaded per lane in each of the Western blots and electrophoretically separated on a 6%, 8%, or 12% gel (depending on the size of the protein of interest). SDS–PAGE gels and all proteins were transferred at 20 V overnight at 4°C to a nitrocellulose membrane (Bio-Rad, Hercules, CA, U.S.A.). Following transfer, the membranes were briefly washed and blocked with 5% non-fat dry milk in TBST (Santa Cruz) at room temperature for 1 h. The blocked membranes were incubated with primary antibodies overnight at 4°C and washed three times with TBST for 10 min each. The membranes were incubated for 1 h at room temperature with the respective appropriate secondary antibody and washed again three times with TBST for 10 min each. The membranes were then incubated for 5 min in Pierce ECL solution (ThermoFisher Scientific, Waltham, MA, U.S.A.) before being visualized with autoradiography films. All primary antibodies were diluted 1/1000-fold, except for Vinculin (1/2000) and GAPDH (1/5000). For xenograft tissue samples, p70 S6 Kinase was diluted at a 1/2000-fold, whereas 4E-BP1 was diluted at a 1/3000-fold. Secondary antibodies were diluted 1/3000-fold. The blots were developed with ECL chemiluminescent substrate. Densitometric analysis was performed using ImageJ to quantify protein band intensities. For each target, normalized protein expression was calculated, and the values were expressed as the ratio of phosphorylated to total protein.

### Immunofluorescence and confocal imaging

CFPAC-1 and HPAF-II cells stably expressing mCherry-EGFP-LC3B were plated onto coverslips (25 mm, 1.5 thickness), placed in a six-well plate at a density of 2 × 10^5^ cells/well and 3 × 10^5^ cells/well, respectively, and cultured at 37°C until reaching ∼70% confluency before treatment. Cells were treated in reduced serum using various treatment conditions: Control, α-MLT 5 mM, HCQ 20 μM, and Combination. Following incubation, cells were washed three times with a buffer consisting of 140 mM NaCl, 5.4 mM KCl, 1.8 mM CaCl_2_, 0.8 mM MgSO_4_, 5 mM glucose, and 25 mM HEPES pH 7.5. Then, cells were incubated for 5 min with 1 μg/ml Hoechst 33342 (Cat# H1399, Molecular Probes) before imaging. Cells were imaged using laser scanning confocal microscopy (Nikon T1-E) with a 100× objective and analyzed with NIS software. Six regions of interest were imaged randomly. The images represent a maximum projection intensity derived from the Z-stack.

### MTT assay and bliss synergy analysis

MTT assay was performed as described previously [12]. The MTT assay is a colorimetric assay used for the quantification of cellular proliferation, viability, and cytotoxicity. The reduction of yellow tetrazolium dye MTT, which is 3-(4,5-dimethylthiazol-2-yl)-2,5-diphenyltetrazolium bromide, to purple formazan crystals is used to assess metabolic activity in the cells. Briefly, cells (1000 cells/well for both CFPAC-1 and HPAF-II) were seeded in 96-well plates and allowed to acclimatize before starting treatments. Afterwards, cells were exposed to two doses of α-MLT (250 μM and 500 μM); HCQ doses ranging from 1, 2.5, 5, 7.5, 10, and 12.5 μM; and respective combinations of HCQ and α-MLT. Cells were incubated at 37°C for 5–6 days with the treatment, and the MTT assay was conducted according to the manufacturer’s instructions. The absorbance was measured at 490 nm with a reference reduction at 655 nm. Following quantification of percent viable cells, Bliss synergy analysis was carried out to see whether the two-drug combinations had antagonistic, additive, or synergistic effects. Synergy scores of *<*−10 were considered antagonistic, −10 to 10 were considered additive, and >10 were considered synergistic [86].

### Colony formation assay

Colony formation assays were performed as previously described [12,13,87]. Briefly, cells were seeded at low density (500 cells/well for CFPAC-1 and 2500 cells/well for HPAF-II) in six-well plates in triplicates. Cells were allowed to acclimatize following treatment. Cells were treated with α-MLT (250 μM and 500 μM), HCQ (7.5, 10, and 12.5 μM), and the combinations of HCQ and α-MLT. Drugs were replenished every 3rd day and were continued for 10 days. At the end of the 10-day period, the medium was removed, and the colonies were washed, fixed with 100% ice-cold methanol, and stained using Enhanced Gram Crystal Violet (Remel). After the colonies were fully dried, they were lysed with lysis buffer containing 0.2 N NaOH in 1% SDS. The absorbance was measured in a spectrophotometer at 595 nm. Graphs were plotted and expressed as colony intensity (% control) versus concentration of drugs.

### Animal welfare and ethical statement

Female athymic nude mice (6-week-old) were purchased from Jackson Laboratories (Bar Harbor, ME, U.S.A.). Animals were housed (5 mice/cage) with *ad libitum* access to food (chow diet) and water. The room was maintained at a temperature of 22°C with a humidity of 40%–60% and a 12:12 h light/dark cycle. Mice were housed in specific pathogen-free zones. Cages were lined with sterilized corncob bedding material. Mice were given ∼7 days to acclimatize to the housing conditions before the start of the experiments. All experimental procedures were in compliance with the National Institute of Health guidelines and approved by the TTUHSC Institutional Animal Care and Use Committee. All studies involving animals are reported in accordance with the ARRIVE guidelines for reporting experiments involving animals. Mice were not deprived of food or water at any time. Efforts were made to minimize animal suffering. All murine experiments were performed in the Laboratory Animal Resource Center at Texas Tech University Health Science Center (TTUHSC) in Lubbock, TX. The Animal Care and Use Program at TTUHSC is fully AAALAC accredited.

### Group size, randomization and blinding

The xenograft studies were performed at least three times, and each group contained five mice (*n* = 5) per experiment. Sample size was determined based on preliminary experiments and prior experience with HPAF-II xenograft studies in our laboratory, which indicated that this group size was sufficient to detect biologically meaningful differences in tumor growth between treatment groups. The mice were selected randomly from the pool of all cages eligible for inclusion in the study and randomly divided into groups. The investigators performing the tumor measurements and outcome analyses were blinded to the group assignment to minimize experimental bias.

### Xenograft studies

Xenograft studies were performed as described previously [11,12,87] at the Laboratory Animal Resource Center at Texas Tech University Health Science Center (TTUHSC) in Lubbock, TX. The Animal Care and Use Program at TTUHSC is fully AAALAC accredited. Six-week-old female athymic nude mice were allowed to acclimatize for about a week before the start of the experiment. Two experimental paradigms were employed, i.e., (1). Prevention model and (2). Treatment model, to evaluate the effects of SLC6A14 inhibition both during tumor initiation and in established tumors. For the experimental procedures, animals were anesthetized using isoflurane in 100% oxygen using a calibrated vaporizer. Induction was done at 3%–4% isoflurane to effect, and animals were maintained at 1.5%–2% isoflurane via nose cone during the procedures. Animals were recovered under direct observation.

#### Prevention model

Here, the mice were subcutaneously injected (100 μl) with 0.5 × 10^6^ of HPAF-II cells that were suspended in serum-free media and Matrigel (1:1 ratio) into both flanks of each mouse. Following injection, the mice were randomly divided into six groups: (1) control, (2) α-MT[DL], (3) HCQ, (4) imipramine, (5) α-MT[DL] + HCQ, and (6) α-MT[DL] + imipramine. Each group consisted of 5 mice. The drugs were provided in drinking water at 2 mg/ml of α-MLT and 0.3 mg/ml of both HCQ as well as imipramine, and the treatment was started on the same day as the cancer cell injection. Imipramine was also included in the study since it is an FDA-approved tricyclic antidepressant and a highly potent inhibitor of macropinocytosis [69].

#### Treatment model

Here, the HPAF-II cell line was injected exactly as described above. The tumors were allowed to grow to ∼100 mm^3^, after which the mice were randomly divided into four groups: (1) control, (2) α-MT[DL], (3) HCQ, and (4) α-MT[DL] + HCQ. The drugs were provided in drinking water at 2 mg/ml of α-MLT and 0.3 mg/ml of HCQ. The combination group received both drugs at the indicated concentrations.

#### Tumor monitoring and tissue collection

In both experimental models, drug-containing drinking water was replenished every 48 h. Tumor growth was measured weekly using a digital caliper, and the tumor volume was calculated using the formula (width^2^ × length)/2. Mouse body weights were also recorded to monitor systemic toxicity. The animals were killed on the 30^th^ day using carbon dioxide asphyxiation in a dedicated chamber using a 30%–70% fill rate followed by cervical dislocation. Euthanasia procedures are compliant with TTUHSC policy and follow current AVMA guidelines. Following extraction, the tumor samples were weighed, and a portion of the tumor tissue was snap-frozen in liquid nitrogen, while the others were embedded, frozen, and stored at −80°C for Western blotting analysis. Some tumors in the treatment model were also injected with TMR Dextran to study macropinocytosis.

### KPC mouse genotyping

Generation of KPC and KPCS mice and the genotyping protocol were described previously [35]. LSL-Kras^G12D/+;^ LSL-p53^R172H/+^ (KP) transgenic mice were bred with Pdx1-Cre mice to get LSL-Kras^G12D/+;^ LSL-p53^R172H/+;^ Pdx1-Cre (KPC) transgenic mice. KPC mice were then treated with or without α-MT[DL] to characterize the induction of autophagy and macropinocytosis. The following PCR genotyping primers were used, as recommended by the Jackson Laboratory: for LSL-Kras^G12D^, wild-type forward: 5ʹ -TGT CTT TCC CCA GCA CAG T-3ʹ, mutant forward: 5ʹ -GCA GGT CGA GGG ACC TAA TA-3ʹ, and common reverse: 5ʹ -CTG CAT AGT ACG CTA TAC CCT GT-3ʹ. For LSL-Trp53^R172H^, wild-type forward: 5ʹ -AGG TGT GGC TTC TGG CTT C-3ʹ, mutant forward: 5ʹ -CCA TGG CTT GAG TAA GTC TGC A-3ʹ, and common reverse: 5ʹ -GAA ACT TTT CAC AAG AAC CAG ATC A-3ʹ. For Pdx-1 Cre, internal positive control forward: 5ʹ -AGA TGG AGA AAG GAC TAG GCT ACA-3ʹ, internal positive control reverse: 5ʹ -CTG TCC CTG TAT GCC TCT GG-3ʹ, transgene forward: 5ʹ -CCT GGA CTA CAT CTT GAG TTG C-3ʹ, and transgene reverse: 5ʹ -AGG CAA ATT TTG GTG TAC GG-3ʹ.

### Statistical analysis

Statistical analysis and graphs were made using GraphPad Prism 8.4.3. Results are expressed as mean ± SEM. Experiments were repeated at least twice or thrice depending on the experiment. Statistical significance was determined using one-way analysis of variance (ANOVA) with a Dunnett’s or Tukey’s post hoc test for multiple comparisons, as well as Student’s *t*-test, and *P*-values have been indicated as follows: NS, non-significant; *P <0.05; **P <0.01; ***P <0.001; and ****P <0.0001.

## Supplementary Material

Supplementary Figure S1

## Data Availability

All datasets generated and/or analyzed during the present study are included in this article.

## Open Access

Open access for this article was enabled by the participation of Texas Technical University Health Sciences Center in an all-inclusive Read & Publish agreement with Portland Press and the Biochemical Society.

## Abbreviations

AATs: amino acid transporters
BafA1: bafilomycin A1
DAPI: 4′,6-Diamidino-2-Phenylindole
EIPA: 5-(N-Ethyl-N-isopropyl)amiloride
HCQ: hydroxychloroquine
KPC: LSL-Kras^G12D/+;^ LSL-p53^R172H/+;^ Pdx1-Cre
mTORC1: mammalian target of rapamycin 1
OCT: optimal Cutting Temperature
PDAC: pancreatic ductal adenocarcinoma
TMR: tetramethylrhodamine
