# Supplementary material for: Dual targeting of SLC6A14 and autophagy/macropinocytosis enhances therapeutic efficacy in pancreatic ductal adenocarcinoma
Source: Biochem J. 2026 Jul 2;483(8):1369–90. doi: 10.1042/BCJ20250155 (PMC13329288; doi:10.1042/BCJ20250155)
Supplement: Supplementary Figure S1 [file BCJ-2025-0155_supp.pdf]

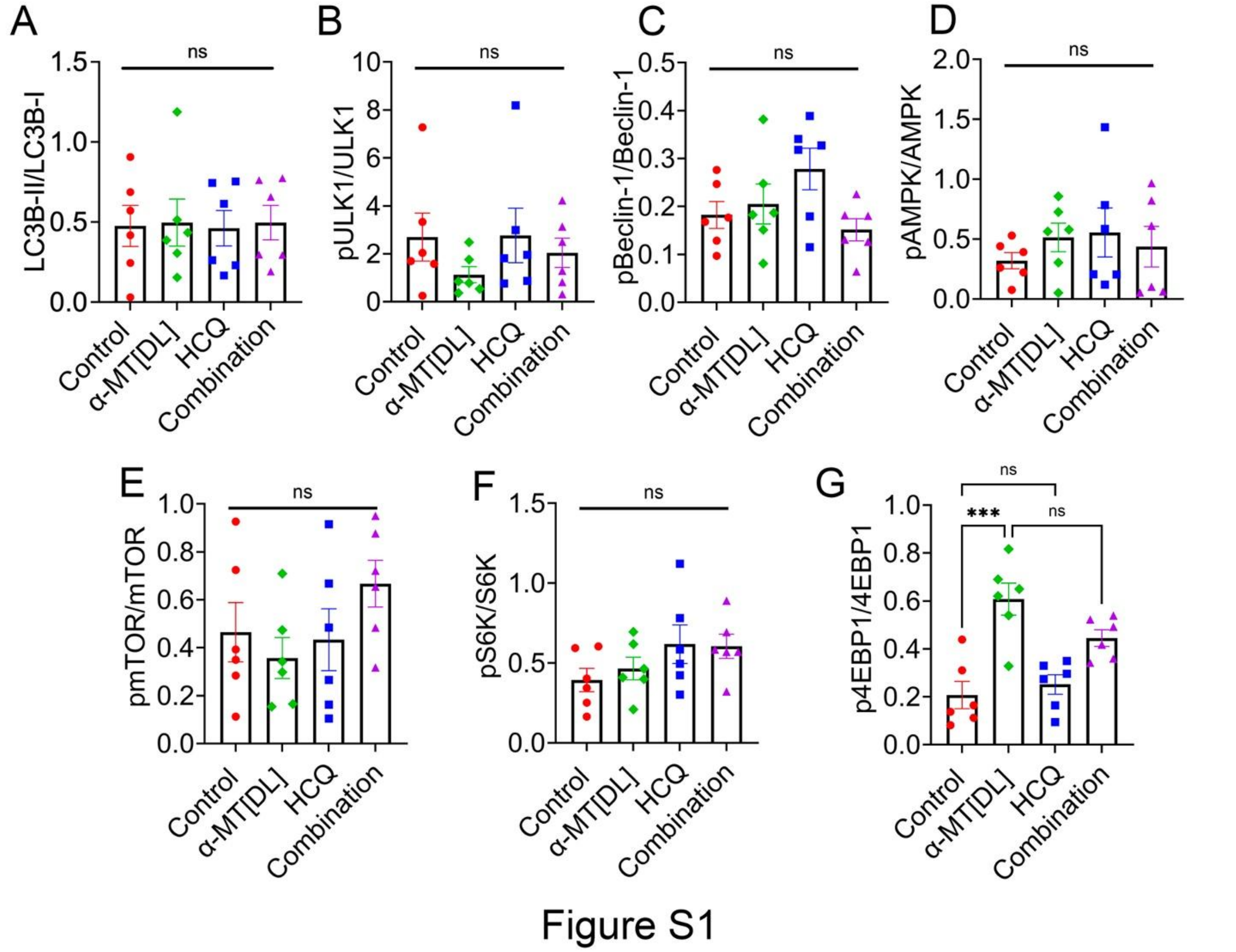

Figure S1. Quantification of LC3B, it upstream target proteins, and mTORC1 downstream effector proteins in the xenograft tumor samples from the control and treatment groups. Densitometric analysis using ImageJ software showing normalized protein expression. Data representated as the (**A**) ratio of LC3B-II/LC3B-I (**B-G**) ratio of phospho/total for all the target proteins of interest – upstream target proteins of LC3B and mTORC1 downstream effector proteins. Data are given as mean ± SEM. ns; non-significant, ***p <0.001.
